# ER proteostasis meets mitochondrial function: contact sites as hubs of communication and therapeutic targets

**DOI:** 10.1111/febs.70431

**Published:** 2026-01-29

**Authors:** Giorgia Maria Renna, Alessandro Cherubini, Ersilia Varone, Serena Germani, Alice Marrazza, Ester Zito

**Affiliations:** ^1^ Istituto di Ricerche Farmacologiche Mario Negri IRCCS Milan Italy; ^2^ Department of Biomolecular Sciences University of Urbino Carlo Bo Italy; ^3^ Department of Molecular and Developmental Medicine University of Siena Italy

**Keywords:** cancer, ERMC, mitochondria metabolism, neuromuscular diseases, proteostasis

## Abstract

Proteostasis maintains the balance between protein synthesis, folding, and degradation within the endoplasmic reticulum (ER). This quality‐control system ensures that proteins undergo proper post‐translational modifications—such as PDI–ERO1‐mediated oxidative folding and STT3‐dependent N‐glycosylation—so that only correctly folded proteins proceed through the secretory pathway. Impairment of protein load, folding capacity, or degradation via the ER‐associated degradation (ERAD) pathway leads to the accumulation of unfolded proteins, triggering ER stress and activating the unfolded protein response (UPR), which, in the first instance, is an adaptive signaling network designed to restore homeostasis by adjusting protein synthesis, enhancing folding capacity, and promoting the clearance of misfolded proteins. During ER stress, the ER undergoes morphological and functional remodeling to manage the increased folding burden, including an increase of ER–mitochondria contact sites (ERMCs). These nanometric junctions (~10–100 nm) facilitate lipid and metabolite exchange and mediate calcium and reactive oxygen species signaling to support cellular metabolism. However, chronic ER stress can further tighten ERMCs, leading to calcium overload, mitochondrial dysfunction, and apoptosis. This review examines the core mechanisms underlying ER proteostasis in the context of ER stress and explores how ER stress first boosts mitochondrial activity and later impairs it through ERMCs, contributing to cell death and disease. Finally, emerging therapeutic strategies aimed at restoring proteostasis and modulating the dynamics of ERMCs are highlighted as promising interventions for conditions, such as cancer and congenital myopathies, where ER and mitochondrial dysfunction play central roles in pathogenesis.

## Graphical abstract

Proteostasis ensures proper protein folding, modification, and degradation, while its impairment triggers ER stress. Chronic ER stress and maladaptive UPR via the CHOP–ERO1 axis remodel ERMCs, altering calcium signaling and mitochondrial metabolism. Tightened contacts and calcium overload lead to mitochondrial dysfunction and apoptosis most likely through the mPTP. Targeting ER proteostasis and ERMCs is a promising therapeutic strategy in diseases whose pathogenesis involves impairment in both proteostasis and ERMCs.

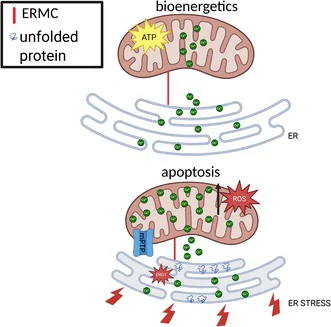

**Abbreviations**
AMFR: autocrine motility factor receptor
ATF4: activating transcription factor 4
ATF6: activating transcription factor 6
BAK: Bcl‐2 Homologous Antagonist Killer
BAX: Bcl‐2‐Associated X protein
BCL‐2: B‐cell lymphoma 2
BiP: Binding Immunoglobulin Protein
CALNX: Calnexin
CHOP: C/EBP homologous protein
CSMDH: Ca^2+^ sensitive matrix dehydrogenases
Drp1: dynamin‐related protein 1
eIF2a: eukaryotic translation initiation factor 2
ER: endoplasmic reticulum
ERAD: ER‐associated degradation
ERdj5: endoplasmic reticulum‐associated degradation and disulfide reductase 5
ERMC: ER–mitochondria contact
ERMCs: ER–mitochondria contact sites for sake of clarity they are also referred as mitochondria‐ER contact sites (MERCs) or mitochondria associated membranes (MAMs)
ERO1: endoplasmic reticulum oxidoreductin 1
ERp44: endoplasmic reticulum resident protein 44
ERp57: endoplasmic reticulum protein 57
GADD34: growth arrest and DNA damage‐inducible protein 34
Grp75: glucose‐regulated protein 75
Grp94: glucose‐regulated protein 94
IP3R: inositol 1,4,5‐trisphosphate receptor
IRBIT: inositol 1,4,5‐trisphosphate receptor (IP3R) binding protein released with inositol 1,4,5‐trisphosphate
IRE1: Inositol‐Requiring Enzyme 1
ISRIB: integrated stress response inhibitor
MFN2: Mitofusin 2
mPTP: mitochondrial permeability transition pore
OMM: outer mitochondrial membrane
OPA1: optic atrophy 1
OST: Oligosaccharyltransferase
PACS‐2: Phosphofurin Acidic Cluster Sorting protein 2
PDI: protein disulfide isomerase
PE: phosphatidylethanolamine
PERK: protein kinase RNA‐like endoplasmic reticulum kinase
PL: phospholipids
PP1C: protein phosphatase 1
PS: phosphatidylserine
PTP: permeability transition pore
ROS: reactive oxygen species
RyR2: ryanodine receptor 2
SEPN1: selenoprotein N
SR: sarcoplasmic reticulum
TCA: tricarboxylic acid
TEM: transmission electron microscopy
TMX1: thioredoxin‐related transmembrane protein 1
TUDCA: Tauroursodeoxycholic acid
UPR: unfolded protein response
VAPB: Vesicle‐Associated Membrane Protein‐Associated Protein B
VDAC1: voltage‐dependent anion channel
VEGFA: vascular endothelial growth factor A
XBP1: X‐box binding protein 1

## Introduction

Proteostasis is central to cellular health, ensuring that proteins are correctly synthesized, folded, and degraded within the endoplasmic reticulum (ER). Impairment of this balance—due to excessive protein load, impaired folding, or defective degradation—leads to the accumulation of misfolded proteins and induces ER stress, which in turn activates the homeostatic unfolded protein response (UPR) [1]. The UPR is initiated by three stress sensors located in the ER membrane: inositol‐requiring enzyme 1 alpha (IRE1α, henceforth IRE1), protein kinase RNA‐like ER kinase (PERK), and activating transcription factor 6 alpha (ATF6α, henceforth ATF6) [2]. A critical pathway of this response involves PERK‐mediated phosphorylation of the translation initiation factor eIF2α, which transiently inhibits global protein synthesis while selectively enhancing the translation of stress‐adaptive genes, most notably ATF4. This branch is a hallmark of the adaptive UPR, designed to reduce the ER's protein load and increase the production of chaperones that promote proper protein folding, thereby restoring proteostasis [3].

This stress response not only aims to restore ER proteostasis but also influences mitochondrial function through physical communication at ER–mitochondria contact sites (ERMCs) [4]. During ER stress, the ER might undergo membrane expansion and structural remodeling, leading to increased contact with mitochondria [5, 6]. These proteinaceous contact sites facilitate the exchange of calcium, lipids, and hydrogen peroxide (H_2_O_2_), all of which are important metabolites for mitochondrial metabolism. For example, ER calcium channels, such as inositol 1,4,5‐trisphosphate receptors (IP3Rs), interact with outer mitochondrial membrane (OMM) proteins, tightening the ER–mitochondria contacts and promoting calcium influx into mitochondria. The ER calcium channels, IP3Rs and RyRs might be redox‐regulated on their luminal side by thioredoxin‐containing chaperones, such as ERp44 and the ER stress‐induced oxidoreductase ERO1α (henceforth, ERO1), thereby promoting calcium release from the ER [7, 8, 9, 10]. Seminal work spanning over three decades has demonstrated that when IP3Rs are retained at ERMC, mitochondrial calcium uptake increases, thereby driving enhanced bioenergetics [6, 11, 12]. Notably, growing evidence indicates that the UPR sensor PERK is enriched at ERMCs, where it contributes to a further regulation between ER proteostasis and mitochondrial function [13]. PERK physically interacts with ERO1 to stabilize ERMCs structure and promote efficient calcium transfer to mitochondria, thereby supporting oxidative phosphorylation and energy production [14, 15]. Altogether, these findings suggest that the ER and adaptive UPR play a central role in calcium signaling and support mitochondrial metabolism through ERMC‐mediated calcium delivery.

Beyond a direct physical communication, ER and mitochondria are also functionally connected through transcriptional regulation. ER stress and downstream UPR induce ATF4, a key metabolic regulator that orchestrates a transcriptional program enabling cells to adapt to proteotoxic stress [16] and maintain mitochondrial function under stress conditions [17].

This direct and functional ER–mitochondria crosstalk not only enhances mitochondrial bioenergetics during the adaptive phase of the UPR but also defines the threshold for stress‐induced apoptosis. Indeed, beyond the initial adaptive mechanism linking ER stress with the boost of mitochondrial activity, excessive calcium influx into mitochondria can trigger apoptosis by inducing the opening of the mitochondrial permeability transition pore (mPTP), initiating cell death pathways [18, 19]. During unresolved chronic ER stress, ATF4 also induces the expression of CHOP (C/EBP homologous protein), a pro‐apoptotic transcription factor. CHOP contributes to mitochondrial dysfunction by upregulating ERO1 and promoting ERO1‐mediated reactive oxygen species (ROS) production, disrupting mitochondrial dynamics, and activating intrinsic apoptotic signaling pathways [20]. These effects are further amplified by CHOP's ability to induce pro‐apoptotic BCL‐2 family proteins and suppress antiapoptotic factors, such as BCL‐2 [21].

In this context, the UPR‐related PERK‐ATF4‐CHOP‐ERO1 axis represents a critical molecular switch that links ER stress and proteostasis to mitochondrial metabolism—initially promoting adaptive metabolic flexibility but ultimately driving mitochondrial dysfunction and cell apoptosis.

Understanding how these signaling pathways coordinate ER–mitochondria communication and fine‐tune the balance between mitochondrial homeostasis and dysfunction offers valuable insights into the molecular basis of diseases characterized by defects in proteostasis (i.e., proteotoxic stress), ERMCs, and mitochondrial impairment, including cancer and congenital myopathies. Such knowledge may also guide the development of effective pharmacological strategies that target both ER stress and ERMCs.

### UPR‐regulated endoplasmic reticulum proteostasis

The ER is a highly specialized organelle composed of an intricate network of interconnected domains, including the smooth ER and the rough ER. The smooth ER is primarily involved in lipid synthesis and carbohydrate metabolism, while the rough ER, characterized by ribosome‐studded membranes, is dedicated to protein translation and folding [22]. In muscle cells, a specialized form of the ER called the sarcoplasmic reticulum (SR) is dedicated to calcium (Ca^2+^) storage and handling [23]. Approximately one‐third of cellular proteins are translocated into the rough ER, where they are folded and processed before entering the secretory pathway. Protein translocation into the ER occurs either co‐translationally—where the nascent polypeptide is translocated during synthesis—or post‐translationally, after synthesis is complete. In both cases, translocation involves the association of the polypeptide with the ER translocon complex, energy‐dependent import into the ER lumen or membrane, and subsequent folding and maturation.

Unfolded proteins are initially recognized by the signal peptidase complex and the oligosaccharyltransferase (OST) complex, which includes the catalytic subunits STT3A and STT3B. OST catalyzes the addition of N‐linked glycans to asparagine residues within the consensus sequence Asn‐X‐Ser/Thr, a critical step for the proper folding of many secreted proteins [24, 25].

Disulfide bond formation, an essential feature of protein maturation, is catalyzed by the PDI–ERO1 electron relay system. PDI introduces disulfide bonds into nascent proteins and is subsequently reoxidized by ERO1, which transfers electrons to molecular oxygen, generating stoichiometric amounts of H_2_O_2_ in proportion to the number of disulfide bonds formed [15]. In addition to enzymatic folding, ER‐resident chaperones—including BiP (an Hsp70 homolog) and the lectins calnexin and calreticulin—bind glycan moieties on nascent proteins to prevent inappropriate folding and aggregation. These chaperones also play a central role in identifying terminally misfolded proteins, which are retro‐translocated into the cytoplasm and targeted for degradation by the proteasome through the ER‐associated degradation (ERAD) pathway [26].

**Fig. 1:**
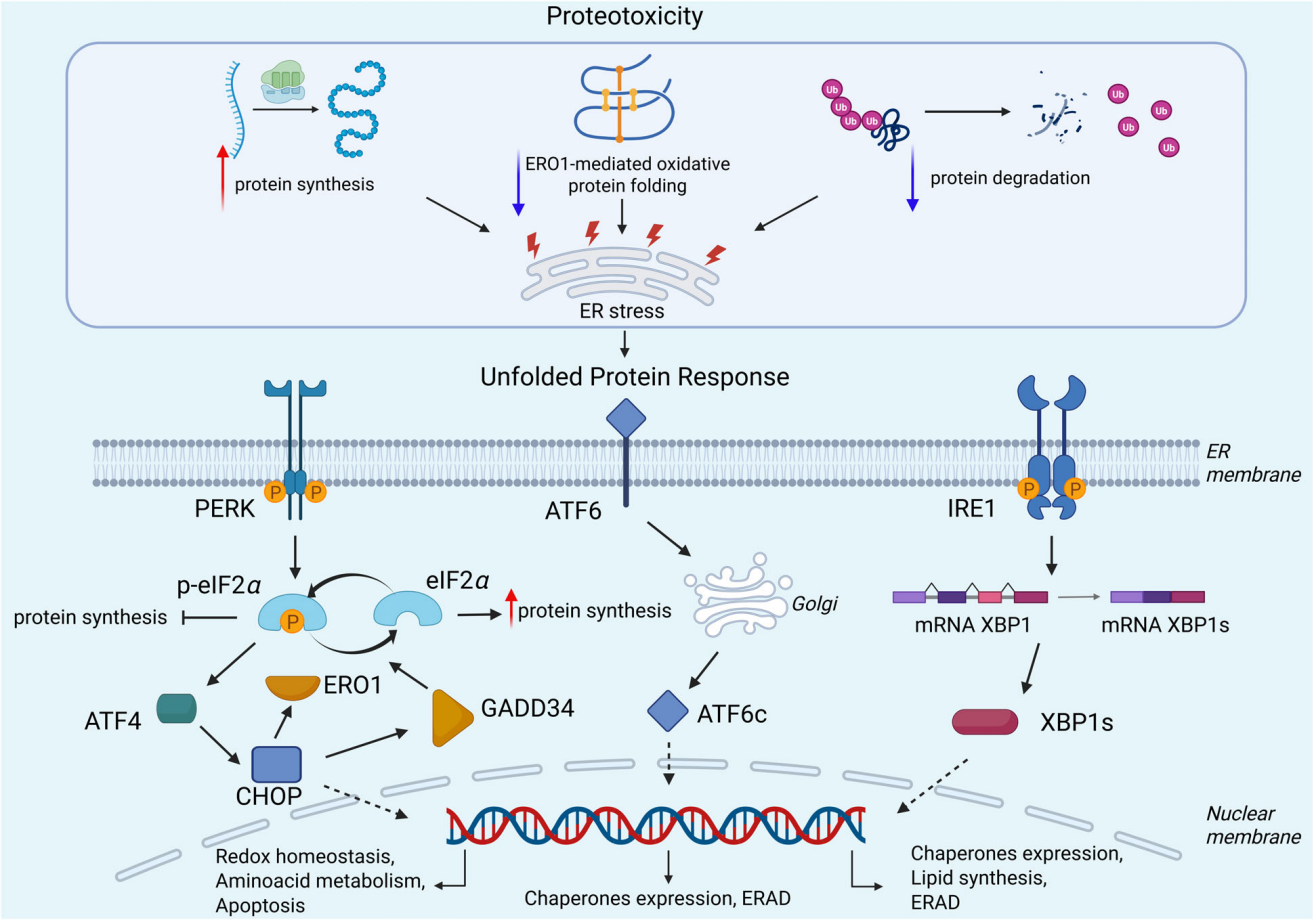
Switch from proteostasis to proteotoxicity triggers endoplasmic reticulum (ER) stress and unfolded protein response (UPR). Proteostasis refers to the balance between protein synthesis, folding, and degradation. An imbalance between these processes leads to proteotoxicity and ER stress due to the accumulation of unfolded proteins. This stress activates the UPR through three ER membrane sensors: protein kinase RNA‐like ER kinase (PERK), activating transcription factor 6 alpha (ATF6), and inositol‐requiring enzyme 1 alpha (IRE1). Activation of PERK leads to phosphorylation of eIF2α, which reduces global protein synthesis. PERK also induces activation of ATF4, a transcription factor involved in amino acid metabolism, redox homeostasis, and apoptosis. ATF4 in turn induces CHOP, which regulates ERO1 (involved in oxidative protein folding) and GADD34 (involved in dephosphorylation of eIF2α to restore protein synthesis). ATF6 activation causes its translocation to the Golgi apparatus, where it is cleaved. The cleaved cytosolic fragment of ATF6 then acts as a transcription factor, promoting the expression of chaperones and components of ERAD. Activation of IRE1 triggers its autophosphorylation and mediates the unconventional splicing of XBP1 mRNA, producing spliced XBP1, a transcription factor that enhances expression of genes involved in protein folding and ERAD.

Conditions that affect protein load, folding, or degradation can lead to the accumulation of unfolded proteins within the ER, triggering a condition known as ER stress. In response, three ER‐resident transmembrane sensors—IRE1, PERK, and ATF6—initiate the UPR, a coordinated signaling network aimed at restoring ER proteostasis [27, 28, 29]. The activation of these UPR sensors primarily depends on the ER chaperone BiP, which under nonstressed conditions binds to their luminal domains, keeping them inactive [30, 31]. When unfolded proteins accumulate, BiP dissociates from the sensors to bind exposed hydrophobic domains of misfolded proteins, thereby allowing UPR activation. This involves oligomerization and autophosphorylation of IRE1 and PERK, and the unmasking of a Golgi‐targeting domain in ATF6 [30, 32, 33]. Upon dimerization and autophosphorylation, PERK phosphorylates the eukaryotic initiation factor eIF2α, leading to a temporary reduction in global protein synthesis. Additionally, this phosphorylation allows for the selective translation of mRNAs containing upstream open reading frames, such as ATF4, a transcription factor that regulates genes involved in amino acid metabolism, redox homeostasis, and apoptosis. ATF4 also induces CHOP, a pro‐apoptotic transcription factor that upregulates GADD34, a regulatory subunit of PP1C, which dephosphorylates eIF2α to resume protein synthesis. CHOP also increases the expression of ERO1, promoting oxidative protein folding while generating H₂O₂ as a by‐product [20, 28]. In the ATF6 branch, upon activation, ATF6 translocates to the Golgi, where it undergoes regulated intramembrane proteolysis. This releases a cytosolic fragment ATF6_c_ that acts as a transcription factor, primarily inducing genes involved in ERAD and chaperone expression. Lastly, IRE1, the most evolutionarily conserved UPR sensor, also dimerizes and autophosphorylates upon activation. This enables its cytosolic RNase domain to splice XBP1 mRNA in an unconventional manner (by removing an intron), producing the active transcription factor XBP1s. XBP1s drives the expression of genes encoding ER chaperones and components of the ERAD pathway [32]. Collectively, the UPR's goal is to restore ER proteostasis through coordinated mechanisms that reduce protein load, enhance folding capacity, and increase degradation of misfolded proteins (Fig. 1). Beyond the ER itself, the UPR also impacts other organelles—most notably mitochondria—through both physical and functional mechanisms. For example, ER stress can lead to ER membrane expansion via increased lipid biosynthesis, which accommodates the higher load of unfolded proteins and strengthens physical contacts with adjacent organelles, such as mitochondria [5, 6]. A key example of ER–mitochondria crosstalk involves the interaction between the UPR sensor PERK and ERO1 at ERMCs, where ERO1‐mediated redox activity regulates mitochondrial dynamics by oxidizing ERMCs‐associated proteins [14]. In addition to these structural connections, a functional relationship exists between the ER and mitochondria. ATF4, for instance, promotes the expression of genes involved in amino acid metabolism, redox balance, antioxidant responses, and mitochondrial one‐carbon metabolism, all of which support mitochondrial function and energy production under stress conditions [17]. Taken together, these observations underscore how impaired ER proteostasis can affect mitochondrial function.

### Calcium handling in ER proteostasis and connection with ERMCs

A fundamental aspect of both maintaining proteostasis and regulating communication between ER and surrounding organelles—particularly mitochondria—is calcium handling. The ER serves as the primary calcium reservoir in the cell, maintaining concentrations ranging from approximately 100 μm to 1 mm. A decrease in ER calcium levels triggers ER stress and activates the UPR, mostly because calcium plays a critical role in regulating molecular chaperones involved in protein folding [34].

The ER hosts several Ca^2+^‐buffering chaperones, including calreticulin, Grp94, BiP, and PDI. Calreticulin is essential for the proper folding and quality control of newly synthesized glycoproteins. Grp94 is a general protein chaperone, while PDI acts on disulfide‐bonded proteins [34]. The UPR regulates the abundance of these chaperones at the transcriptional level to restore proteostasis [35]. However, this process operates on a slower timescale due to its dependence on transcription. In contrast, ER Ca^2+^ levels may serve as a rapid means to modulate the function of these Ca^2+^‐buffering chaperones, enabling the cell to quickly adapt to acute increases in unfolded protein load [36].

ER Ca^2+^ levels are maintained by the sarco/endoplasmic reticulum calcium ATPase (SERCA), an ATP‐dependent pump that imports calcium into the ER [37]. Additionally, Ca^2+^ efflux into the cytosol is mediated by IP3Rs and ryanodine receptors (RyRs) [38]. The activity of these three key Ca^2+^‐handling proteins—and ER Ca^2+^ levels more broadly—are intricately linked to and mutually influenced by the ER redox environment, highlighting the fine‐tuned connection between Ca^2+^ handling and redox proteostasis.

Upon ER Ca^2+^ depletion, the ER becomes more reduced, which likely affects the activity of ER oxidoreductases, such as PDI and Selenoprotein N (SEPN1), ultimately impairing oxidative protein folding and altering the overall redox status of ER‐resident proteins [39, 40]. In addition, the redox status of specific cysteine residues in SERCA, IP3Rs, and RyRs fine‐tunes their function and is regulated by several oxidoreductases. For example, luminal disulfide bonds in SERCA can be reduced by Erp57, Erdj5, or SEPN1, enhancing ER calcium uptake [37, 40, 41, 42]. In contrast, oxidoreductases such as ERO1 and TMX1 promote oxidation and inactivation of SERCA [43, 44] while ERO1 also facilitates oxidation and activation of IP3Rs [8] and RyR2 [10]. Additionally, the thioredoxin‐containing chaperone Erp44 has been shown to inhibit both IP3R and RyR2 activity [9, 10]. Furthermore, oxidation of regulatory cysteines by ERp46 activates IP3Rs, whereas reduction by ERdj5 inhibits their activity [42].

**Fig. 2:**
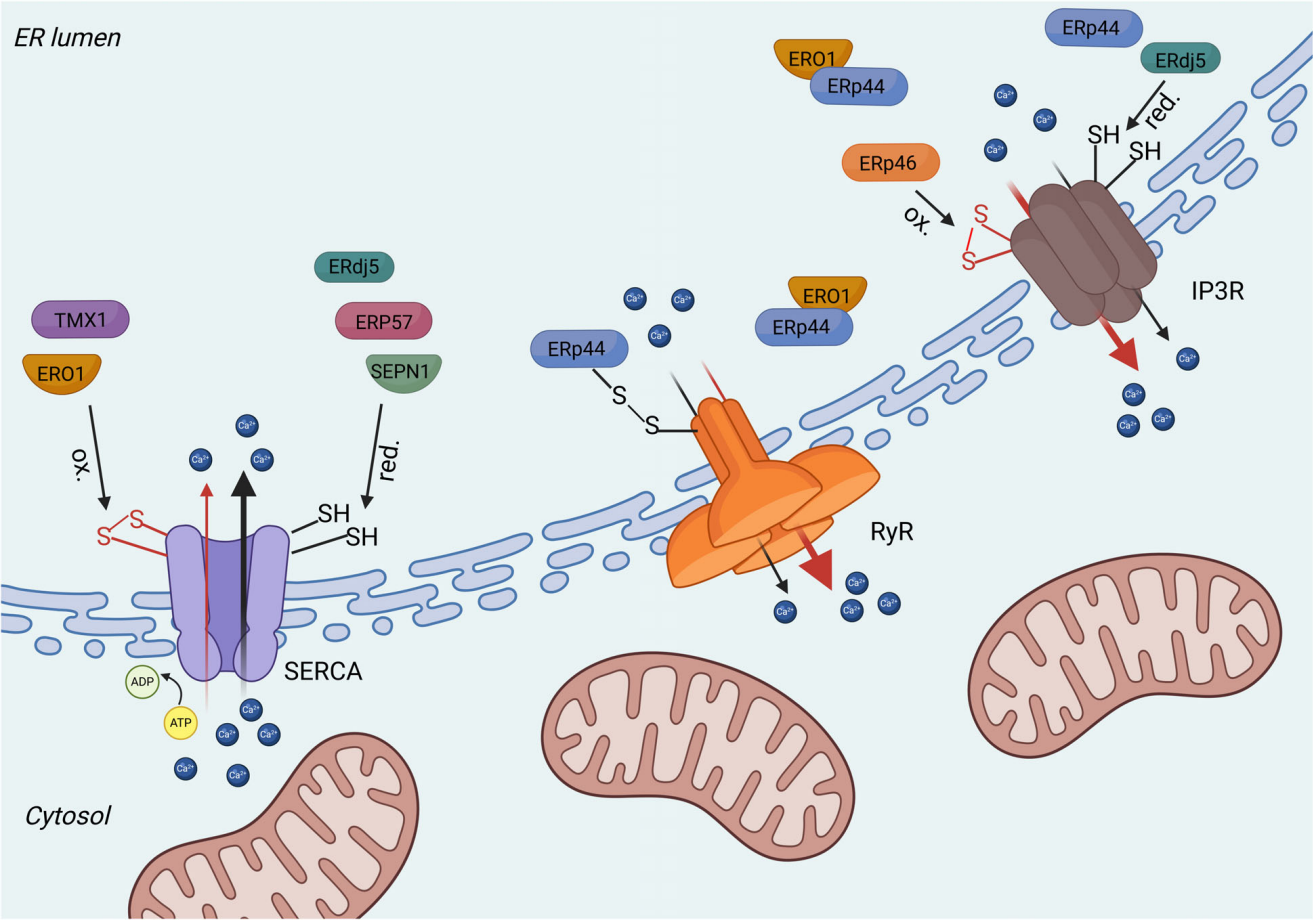
Redox regulation of calcium‐handling proteins at the endoplasmic reticulum (ER)–mitochondria interface. The ER/sarcoplasmic reticulum (SR) plays a central role in calcium homeostasis by regulating calcium flux through several key proteins, including multiple isoforms of sarco/endoplasmic reticulum calcium ATPase (SERCA) (SERCA1, SERCA2, and SERCA3), ryanodine receptor (RyR) (RyR1, RyR2, and RyR3), and inositol 1,4,5‐trisphosphate receptors (IP3R) (IP3R1, IP3R2, and IP3R3). Their activities are finely tuned by redox modifications targeting specific cysteine residues. SERCA exists in several isoforms with tissue‐specific expression: SERCA1a and 1b are primarily found in fast‐twitch skeletal muscle, SERCA2a predominates in cardiac muscle, and SERCA2b is widely expressed in smooth muscle and nonmuscle cells. Redox regulation of two cysteines in the L4 domain of SERCA2b enhances pump activity when are reduced by ERp57, SEPN1, or the protein disulfide isomerase ERdj5, promoting calcium uptake into the ER (red. stands for reduction and ox. for oxidation). Conversely, oxidation of these cysteines by ERO1 and TMX1 diminishes SERCA2b activity. RyR is a tetrameric calcium release channel with multiple isoforms, each showing tissue‐specific expression: RyR1 is mainly present in skeletal muscle, whereas RyR2 is enriched in cardiac muscle. RyR2 activity is regulated by redox modifications of key cysteine residues. Increased ERO1 levels decrease ERp44‐mediated regulation, resulting in elevated RyR2‐dependent calcium release. IP3Rs are also tetrameric calcium release channels and are fundamental mediators of calcium signaling in cells. Cysteines in the third luminal loop of IP3R can bind ERp44, inhibiting calcium efflux. Recent findings reveal that four cysteine residues in the ER lumen are critical for IP3R function: two are essential for tetramer formation, while the other two regulate channel activity via redox modifications. Oxidation of these regulatory cysteines by ERp46 activates IP3Rs, whereas reduction by ERdj5 inhibits their activity.

Notably, many of these oxidoreductases (e.g., SEPN1, ERO1, and TMX1) [44, 45, 46] and calcium‐handling proteins (e.g., SERCA, IP3Rs, and RyRs) [11, 47] are enriched at ERMCs, highlighting their critical role in redox modulating calcium signaling at the ER–mitochondria interface (Fig. 2).

### ERMCs

Ultrastructural studies using transmission electron microscopy (TEM) have established that the ER forms proteinaceous nanometric contacts (10–100 nm) with adjacent mitochondria [6, 48]. Although the distance between the two organelles can be less than 10 nm [6], the large cytoplasmic domains of ER/SR Ca^2+^ release channels—such as IP3Rs and RyRs—protrude approximately 10–12 nm from the ER membrane surface [49, 50], thereby setting a physical limit that prevents the ER and mitochondria from approaching closer than ~10 nm. The distance between the ER and the OMM, which defines the length of ERMCs, can vary from tight contacts of ~10–20 nm to looser contacts extending up to ~100 nm [6]. The heterogeneous lengths (10–100 nm) of ERMCs suggest that several proteins are involved in linking the two organelles, thereby contributing to their variable spacing [6]. These contacts can form either through the direct binding of proteins between opposing membrane surfaces (tethers) or via a single ‘linker’ protein that contains two membrane‐interacting domains, which stabilize membrane subdomains and facilitate inter‐organelle communication. Another group of proteins functions as promoters or disruptors of ERMCs, acting either locally at the organellar interface or indirectly from a distance [51]. Over the years, a growing number of ER and mitochondrial proteins have been identified as components of ERMCs [51]. These include, among others, the bipartite ER–mitochondria tether mitofusin 2 (MFN2) [52], the contact promoters autocrine motility factor receptor (AMFR) [53] and PACS‐2, as well as the ER‐resident proteins TMX and calnexin (CALNX) [54]. For a comprehensive list of ERMCs‐associated proteins, we refer the reader to [51].

The first described function of these contacts was the exchange of phospholipids (PLs) between the organelles [55]. For example, phosphatidylserine (PS), synthesized in the ER by PS synthase‐1 and PS synthase‐2 (enriched at ERMCs), is transferred to the mitochondrial inner membrane, where it is converted into phosphatidylethanolamine (PE) [56, 57]. Proteinaceous tethers between the ER and mitochondria are essential not only for maintaining contact but also for enabling Ca^2+^ transfer. Indeed, Ca^2+^ transfer is blunted by proteolytic treatment [6] and can be restored using synthetic ER–mitochondria linkers [58]. The importance of Ca^2+^ nanodomains for mitochondria Ca^2+^ uptake at the ER–mitochondria interface lies in the fact that the mitochondrial Ca^2+^ uniporter (mtCU) on the inner mitochondrial membrane remains closed at resting cytoplasmic Ca^2+^. Activation of the uniporter requires Ca^2+^ concentrations higher than cytoplasmic peaks, which are achieved at ER/SR–mitochondria contacts where IP3R‐ and RyR‐mediated Ca^2+^ nanodomains are formed [59]. The ERMCs are formed between IP3Rs on the ER membrane which tether to voltage‐dependent anion channel (VDAC1) on the OMM through the chaperone glucose‐regulated protein 75 (GRP75) [60]. IRBIT, an IP3R‐binding protein, promotes ERMC formation in a phosphorylation‐dependent manner [61]. In striated muscle, RyRs located on the SR at the terminal cisternae form SR–mitochondria contacts [47] via MFN2 [62] and the linker protein VAPB [63]. In the OMM, VDAC1 facilitates Ca^2+^ transfer from both IP3R [64] and RyR2‐derived Ca^2+^ signals [65]. For effective communication, the ER/SR Ca^2+^ release channels and OMM porins (VDACs) must be within ~100 nm of each other. However, recent studies highlight that the clustering of IP3Rs at ERMCs mediates calcium transfer between the ER and mitochondria without the need for pore‐forming proteins [11].

Importantly, ER/SR–mitochondria Ca^2+^ transfer depends not only on IP3R and RyR activity but also on the function of SERCA pumps at ERMCs, and thus, on the overall ER calcium content [66]. Several mechanisms regulate SERCA activity. For instance, the chaperone CALNX inhibits SERCA activity in a phosphorylation‐dependent manner [67], although later studies also suggested CALNX as a positive regulator of SERCA2b [68]. Thioredoxin‐containing proteins TMX1 and SEPN1, both enriched at ERMCs, interact with SERCA2b in a redox‐dependent manner, inactivating or activating it, respectively [40, 44]. Notably, TMX1 and SEPN1 also promote ERMC formation, suggesting that modulating SERCA2b activity at ERMCs may regulate mitochondrial Ca^2+^ uptake [45, 69]. Another MAM‐resident redox regulator, the ER transmembrane protein glutathione peroxidase 8 (GPX8), also inhibits SERCA2b activity [70].

**Fig. 3:**
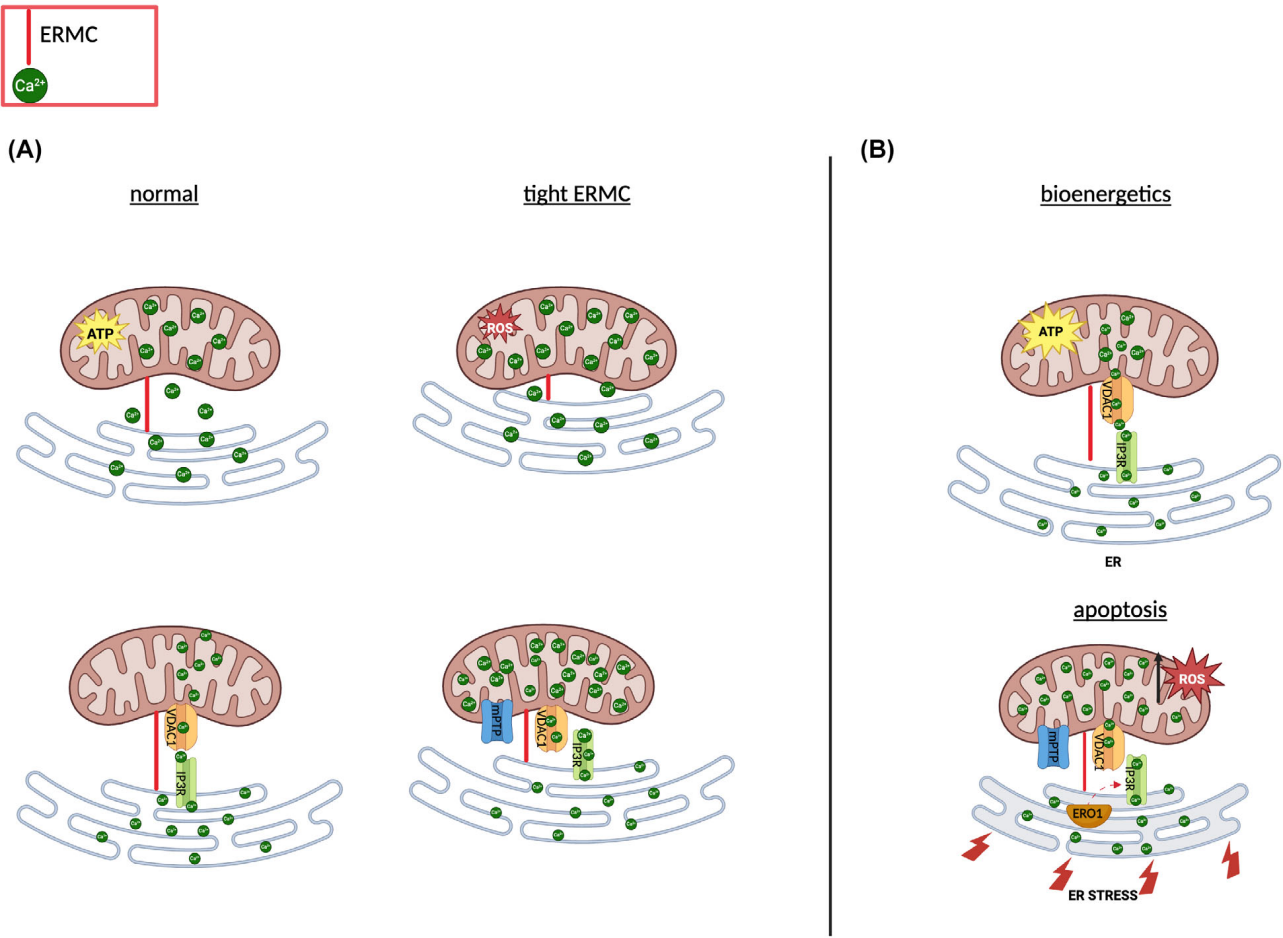
Endoplasmic reticulum (ER)–mitochondria crosstalk governs the balance between cell survival and apoptosis. (A) The physical distance between ER and mitochondria plays a critical role in regulating calcium transfer between these organelles. A close ER–mitochondria apposition improves calcium uptake into the mitochondria through specialized contact sites known as ERMCs. IP3Rs at the ER membrane interact with voltage‐dependent anion channel (VDACs) at the outer mitochondrial membrane, a connection that could be mediated by glucose‐regulated protein 75 (GRP75). Controlled IP3R‐mediated calcium release supports mitochondrial function and cell survival by sustaining metabolism and signaling pathways. Tight ER–mitochondria contact sites (ERMCs) or excess of inositol 1,4,5‐trisphosphate receptors (IP3R) activity at ERMCs can cause mitochondrial calcium overload, triggering the mitochondrial permeability transition pore (mPTP). Thus, IP3R acts as a molecular switch that integrates cellular signals to determine cell fate through its regulation of ER–mitochondria calcium dynamics. (B) Under physiological conditions, controlled calcium efflux from the ER to mitochondria through ERMCs supports essential cellular functions. Moderate mitochondrial calcium uptake stimulates the tricarboxylic acid (TCA) cycle and enhances ATP production through oxidative phosphorylation, thereby promoting mitochondrial bioenergetics and supporting cell survival. In contrast, during chronic ER stress—triggered by the accumulation of misfolded proteins—calcium signaling becomes dysregulated. For example, enhanced expression and activity of ERO1 lead to hyperactivation of IP3Rs. This results in excessive and sustained calcium efflux from the ER into mitochondria via ERMCs. Mitochondrial calcium overload disrupts mitochondrial membrane potential and promotes the opening of mPTP, ultimately triggering the release of apoptogenic factors, such as cytochrome c. These events lead to activation of the intrinsic apoptotic pathway.

IP3Rs at ERMCs—and the ERMCs themselves—are both necessary and sufficient for ER‐to‐mitochondria calcium transfer. However, excessive IP3R activity or overly tight ER–mitochondria contacts can lead to cell death. For example, the interaction of ERO1 with PERK at ERMCs supports mitochondrial metabolism, whereas the C/EBP homologous protein (CHOP) drives calcium‐dependent apoptosis through an ERO1–IP3R‐mediated pathway under ER stress [8]. Likewise, studies using synthetic linkers to artificially enforce extremely tight contacts (< 5 nm) demonstrate that such constricted ERMCs predispose cells to mitochondrial calcium overload, permeability transition, and apoptotic cell death [6]. Together, these findings indicate that ER stress, the CHOP–ERO1 axis, IP3R activity, and the nanoscale spacing between the ER and mitochondria collectively shape cell fate—tuning the balance between adaptive survival pathways and maladaptive, pro‐apoptotic responses [6, 8] (Fig. 3 A,B).

### Mitochondria

Mitochondria are often referred to as the powerhouses of the cell due to their essential role in ATP production through oxidative phosphorylation. Rather than existing as isolated, static structures, mitochondria form a dynamic and interconnected network that constantly undergoes morphological changes via the two processes of fusion and fission. These events are tightly regulated and crucial for maintaining mitochondrial function, distribution, and quality control. Mitochondrial fission (i.e., division) is not a random event but is orchestrated by multiple factors, including physical interactions between the ER and mitochondria at specialized contact sites known as ERMCs. These sites mark mitochondrial constriction points, where proteins such as dynamin‐related protein 1 (Drp1) are recruited to complete the fission process [71]. However, excessive or dysregulated mitochondrial fission—often linked to altered ER–mitochondria interactions—can impair mitochondrial bioenergetics, reducing ATP production and increasing oxidative stress. Thus, beyond the shape and proximity of the ER and mitochondria, their motility also favors their docking.

ERMCs also play a key role in calcium (Ca^2+^) signaling between the ER and mitochondria, contributing to metabolic regulation. Calcium efflux from the ER via IP3Rs and RyRs leads to mitochondrial Ca^2+^ uptake at these contact sites. This, in turn, stimulates Ca^2+^‐sensitive matrix dehydrogenases (CSMDH) in the tricarboxylic acid (TCA) cycle, thereby enhancing mitochondrial metabolism and ATP production [72, 73]. Thus, ER–mitochondria contacts not only serve as platforms for mitochondrial division but also play a crucial role in regulating mitochondrial function and maintaining cellular energy homeostasis, acting as conduits for Ca^2+^ and other metabolites.

These contacts are also central to the cellular switch from an energetic state to an apoptotic phenotype (Fig. 3B). When mitochondrial Ca^2+^ exceeds a critical threshold, it can trigger the opening of the mPTP, allowing the uncontrolled exchange of ions and small solutes between the mitochondrial matrix and the cytosol. PTP opening results in dissipation of the mitochondrial membrane potential, mitochondrial swelling, and the release of pro‐apoptotic factors from the intermembrane space (IMS), initiating the apoptotic cascade. PTP opening is thought to occur when Ca^2+^ uptake is extremely rapid or when mitochondria accumulate large amounts of Ca^2+^ [74].

A critical link between Ca^2+^ signaling, apoptosis, and ER stress/UPR is mediated by the BCL‐2 family of proteins, which includes both pro‐apoptotic (such as BAX and BAK) and antiapoptotic members [75]. The BCL‐2 family plays a pivotal role in coordinating ER–mitochondria crosstalk and regulating apoptosis, particularly under conditions of chronic or irreversible ER stress. During sustained ER stress, activation of UPR sensors leads to the induction of ATF4 and CHOP, which promote the transcriptional upregulation of BIM, a pro‐apoptotic BH3‐only protein. BIM facilitates the oligomerization of BAX and/or BAK at the OMM, resulting in the release of cytochrome c and activation of caspase‐dependent apoptosis. Concurrently, CHOP also represses the expression of antiapoptotic BCL‐2 [76]. BCL‐2 family members also regulate calcium homeostasis at the ER by modulating IP3R activity. Thus, the BCL‐2 family enables the regulation of apoptosis at multiple levels and through different pathways—ranging from the direct control of OMM permeability and the UPR [77] to the modulation of intracellular Ca^2+^ homeostasis.

### The ER stress/proteotoxic stress–UPR–ERMCs–mitochondria axis in diseases and its pharmacological targeting

Alterations in ERMC architecture or signaling have been implicated in a broad spectrum of diseases, including neurodegenerative disorders [78], metabolic syndromes, such as insulin resistance and nonalcoholic fatty liver disease (NAFLD) [79], inflammatory conditions [80], cancer, and muscular pathologies, such as congenital myopathies. In the following sections, we focus specifically on cancer and congenital myopathies, which have been the central focus of our research over the past decade.

### Cancer

Cancer cells are exposed to proteotoxic and ER stress, primarily due to oncogenic mutations that increase protein synthesis, as well as environmental factors, such as hypoxia (i.e., low oxygen levels), which impairs the formation of post‐translational disulfide bonds [81], and limited nutrient availability, which restricts protein synthesis. Additionally, the resulting UPR, primarily mediated by the sensors IRE1 and PERK, plays well‐established roles in cancer progression, and several components of ERMCs—including PML, TMX, and ERO1—function as either tumor suppressors or promoters, depending on the cellular context [44, 82, 83, 84, 85, 86, 87, 88]. Furthermore, alterations in mitochondrial metabolism, particularly the shift from glycolytic to oxidative metabolism, are well‐characterized drivers of cancer aggressiveness [89]. Together, these observations highlight ER stress and altered proteostasis, the UPR, ERMCs, and mitochondrial metabolic reprogramming as key mechanisms driving cancer development and progression. They also suggest that targeting proteostasis may reduce cancer resilience by disrupting downstream processes, such as ERMCs function and mitochondrial metabolic reprogramming.

ERO1, through its mechanism of disulfide bond oxidase of newly synthesized client proteins, supports proteostasis [90]. More recently, ERO1 has been implicated in the regulation of ERMCs contacts, through oxidation of proteins resident at ERMCs, and mitochondrial metabolism [14]. In the ERO1‐dependent electron transfer cascade, molecular oxygen acts as the terminal electron acceptor, ultimately being converted into hydrogen peroxide (H_2_O_2_)—a ROS that, when accumulated in excess, can lead to oxidative stress and maladaptive cellular outcomes [15]. The H_2_O_2_ generated by ERO1 activity is normally buffered by PRDX4, an ER‐resident peroxidase that links H_2_O_2_ detoxification to the re‐oxidation of PDI. This feedback loop not only limits harmful H_2_O_2_ buildup but also sustains oxidative protein folding, which may explain the relatively mild phenotypes observed in ERO1 loss‐of‐function models in mammals [91, 92, 93, 94]. Notably, ERO1 is frequently overexpressed in tumors relative to adjacent healthy tissue, implicating it in cancer pathophysiology [84]. Over the years, our studies have focused on the role of ERO1 in breast cancer progression and aggressiveness. ERO1 function becomes critical under hypoxic conditions—typical of solid tumors. Indeed, genetic deletion of ERO1 in breast tumors blunts VEGFA signaling at multiple levels by impairing its oxidative folding and N‐glycosylation [85, 95]. This inhibits angiogenesis and reduces tumor aggressiveness and therapy resistance [86, 95].

Notably, simultaneous inhibition of VEGF signaling—through a VEGFA monoclonal antibody—or activation of protein synthesis through the small molecule ISRIB [96], when combined with genetic deletion of ERO1, significantly impairs breast tumor resilience by impairing tumor proteostasis [85, 86]. The limited impact of ERO1 loss in healthy tissues, coupled with its upregulation and functional involvement in cancer, makes it an attractive pharmacological target for cancer therapy. To this end, we have recently shown that novel ERO1 inhibitors targeting the catalytic cysteine residue (Cys397) of ERO1 demonstrate promising potential as treatments for breast cancer [83, 97]. These findings suggest that targeting ERO1 may exert anticancer effects at multiple levels by disrupting proteostasis, impairing ERMCs function, and finally impairing mitochondrial metabolism and rewiring.

### Congenital myopathies

ER stress is emerging as a key pathogenic mechanism in many congenital myopathies [98], suggesting that its pharmacological targeting may offer therapeutic benefits [99]. However, the molecular mechanisms driving the switch from adaptive to maladaptive UPR—ultimately resulting in a pathological muscle phenotype—remain to be fully elucidated [98].

SEPN1 is a type II ER membrane protein that functions as a calcium sensor through redox modulation of SERCA activity [40]. Loss of SEPN1 function causes SEPN1‐related myopathy (SEPN1‐RM), a rare congenital myopathy (SEPN1‐RM) [41]. An overlapping muscle phenotype is observed in RYR1‐related myopathy (RYR1‐RM), caused by the I4898T mutation in the SR‐localized RYR1 calcium release channel—one of the most common mutations in RYR1—which impairs ER calcium release [100]. Both SEPN1‐RM [101] and the I4895T RyR1 knock‐in mouse model (the murine equivalent of the human I4898T mutation) [100, 102] are associated with ER stress, impaired ERMCs structure, and mitochondrial function. Treatment with chemical chaperones has shown beneficial effects in both models—TUDCA in SEPN1‐RM and 4‐PBA in the I4895T RyR1 knock‐in mouse [100, 101]. Chemical chaperones are small molecules designed to restore proper protein folding and support ER proteostasis. For this reason, they are commonly referred to as pan‐ER stress inhibitors [103]. Our data show that a 3‐week treatment with TUDCA in SEPN1 knockout (KO) mice—or, alternatively, genetic deletion of ERO1—improves muscle phenotype by enhancing ER proteostasis, calcium handling, ERMCs integrity, and mitochondrial bioenergetics [101]. These findings support our hypothesis that chemical chaperones like TUDCA not only restore ER proteostasis but also reprogram ER function to improve calcium handling via ERMCs, thereby enhancing mitochondrial function and bioenergetics [103, 104]. Consistently, genetic deletion of ERO1 in SEPN1 KO mice mimics the effects of TUDCA, suggesting that ERO1 inhibitors may offer an alternative therapeutic strategy for SEPN1‐RM through a similar mechanism of action [97, 105]. Recently, I29, a novel pyrazolone‐based ERO1 inhibitor, increases mitochondrial membrane potential in SEPN1‐deficient muscle cells, suggesting improved mitochondrial activity [97].

## Conclusion

In this review, we have discussed the molecular mechanisms linking proteostasis, ER stress, and the UPR with mitochondrial function via ERMC interface. Impaired proteostasis—particularly alterations in calcium signaling through ERMCs—modulates mitochondrial bioenergetics. While moderate ER stress may promote cellular adaptation, chronic ER stress, surpassing a calcium‐dependent threshold, activates apoptotic pathways via the mitochondria. An intriguing question, which remains an object of future investigation, is how ERO1 associated with ERMCs switches from a booster of mitochondrial bioenergetics [14] to acting as a maladaptive trigger linked to disease [8, 101]. In the context of disease, we have highlighted the critical role of ERMCs in both cancer and congenital myopathies, diseases that have been a central focus of our recent research. Despite their emerging importance, no direct pharmacological tools are currently available to specifically target ERMCs, aside from synthetic ER–mitochondria linkers, whose therapeutic utility remains uncertain and unproven in clinical settings [6].

We propose that strategies aimed at restoring proteostasis—such as the use of chemical chaperones or ERO1 inhibitors—may also help re‐establish ERMC function and mitochondrial bioenergetics. These approaches could hold therapeutic potential for diseases characterized by impaired proteostasis, ERMCs dysfunction, and compromised mitochondrial metabolism.

Finally, a bidirectional mode of ERMC‐mediated communication is also conceivable. Mitochondria can transmit retrograde signals to the ER via ERMCs—for example, through the production of H_2_O_2_, which oxidizes ERMC‐associated proteins [106], or through changes in ATP levels that regulate protein folding and ER homeostasis. A paradigmatic example of such retrograde signaling is the ablation of Optic Atrophy 1 (OPA1), an inner mitochondrial membrane protein, which induces a premature muscle‐aging phenotype associated with ER stress [107]. This dynamic, two‐way communication between the ER and mitochondria suggests—and warrants further investigation—that therapeutic strategies aimed at restoring ER proteostasis may also be effective in treating mitochondrial diseases via the ERMC interface.

## Conflict of interest

The authors declare no conflict of interest.

## Author contributions

GMR, AC, EZ, SG, AM, and EV contributed to the conception and design of the study. GMR, AC, and EZ drafted the manuscript. EZ secured the funding.
